# Evaluation of the Comet Assay for Assessing the Dose-Response Relationship of DNA Damage Induced by Ionizing Radiation

**DOI:** 10.3390/ijms141122449

**Published:** 2013-11-14

**Authors:** Yan Wang, Chang Xu, Li Qing Du, Jia Cao, Jian Xiang Liu, Xu Su, Hui Zhao, Fei-Yue Fan, Bing Wang, Takanori Katsube, Sai Jun Fan, Qiang Liu

**Affiliations:** 1 Institute of Radiation Medicine, Chinese Academy of Medical Sciences and Peking Union Medical College, Tianjin 300192, China; E-Mails: bamboo201306@163.com (Y.W.); xuchang2001@yahoo.com (C.X.); duliqing2004@126.com (L.Q.D.); jillpumc@gmail.com (J.C.); fanfeiyue@irm-cams.ac.cn (F.-Y.F.); fansaijun@gmail.com (S.J.F.); 2 Tianjin Key Laboratory of Molecular Nuclear Medicine, First Clinical Department of Medical Emergency Response Center for Nuclear Accidents, Ministry of Health, Tianjin 300192, China; 3 National Institute for Radiological Protection and Nuclear Safety, Chinese Center for Disease Control, Beijing 100088, China; E-Mails: jxlamy@gmail.com (J.X.L.); suxu@nirp.cn (X.S.); 4 Medical Emergency Response Center for Nuclear Accidents, Ministry of Health, Beijing 100088, China; 5 Tianjin Key Laboratory of Food and Biotechnology, Tianjin University of Commerce, Tianjin 300134, China; E-Mail: zhaohui@tjcu.edu.cn; 6 National Institute of Radiological Sciences, Chiba 263-8555, Japan; E-Mails: jp2813km@nirs.go.jp (B.W.); tkatsu@nirs.go.jp (T.K.)

**Keywords:** radiation, dose-response relationship, DNA damage, comet assay

## Abstract

Dose- and time-response curves were combined to assess the potential of the comet assay in radiation biodosimetry. The neutral comet assay was used to detect DNA double-strand breaks in lymphocytes caused by γ-ray irradiation. A clear dose-response relationship with DNA double-strand breaks using the comet assay was found at different times after irradiation (*p* < 0.001). A time-response relationship was also found within 72 h after irradiation (*p* < 0.001). The curves for DNA double-strand breaks and DNA repair *in vitro* of human lymphocytes presented a nice model, and a smooth, three-dimensional plane model was obtained when the two curves were combined.

## 1. Introduction

According to data reported by Turai [1], 134 deaths were registered after 420 radiation accidents worldwide between 1944 and 2002. This number is certainly not the total, as it is likely that some accidents worldwide (in particular those related to military or therapeutic use of radiation sources) were not registered by organizations such as the International Atomic Energy Association (IAEA) or the World Health Organization (WHO).

Since the “9/11” terrorist attack in America, people have become increasingly concerned that radiation sources may be utilized by terrorists [1,2]. Additionally, the potential risk of radiation accidents is increasing, especially in developing or politically disintegrating countries [3]. The effective medical management of radiation overexposure incidents requires appropriate estimation of the biological dose to aid the treating physician. Classical cytogenetic biodosimetry analysis includes chromosome aberration (CA) and micronucleus (MN) assays [4]. The chromosome aberration assay introduced by Bender and Gooch [5] remains the “gold standard” for early-response accident biodosimetry and definitive dose assessment [6], but it is time-consuming for the purpose of rapid response because the lymphocyte culturing process takes approximately 48 h to 72 h. Therefore, rapid and sensitive methods are needed to assess the DNA damage induced by ionizing radiation.

The comet assay, also known as single-cell gel electrophoresis, which can detect DNA damage and the repair kinetics at the level of a single cell, has been widely used in radiation biology, toxicology, oncology, and molecular epidemiology in recent years [7–13]. The comet assay is a rapid and sensitive microdosimetric technique that may be suitable for *in vivo* human biomonitoring, especially in cases of incidental exposure to ionizing radiation [14–16]. To fit the dose- and time-response curves after *in vitro* radiation, the difference in the number of DNA double-strand breaks (DSBs) in lymphocytes between *in vivo* and *in vitro* radiation exposures should be studied first.

## 2. Results and Discussion

### 2.1. Differentiation of Apoptotic and Comet Cells

**Figure 1:**
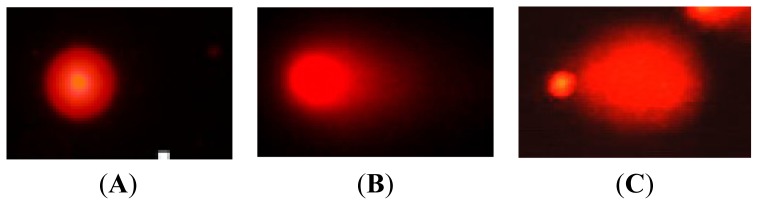
Comparison of control, comet, and apoptotic cells based on fluorescence microscopy [(**A**): Normal cell, a round cell without a tail; (**B**): Comet cell, a cell that contains a tail and a head like a comet; and (**C**): Apoptotic cell, a cell with a large tail and a small head)].

Peripheral blood lymphocytes (PBLs) are known to be very radiosensitive, and they readily undergo apoptosis, with some cells being affected 24 h after irradiation. This radiosensitivity could have greatly affected the comet assay results. Data from those apoptotic cells would not represent radiation-induced DSBs. Apoptotic cells were differentiated from comet cells in the present study according to the appropriate guidelines [17,18] (Figure 1). We did not include apoptotic cells when we scanned for comet cells under a fluorescence microscope.

### 2.2. *In Vivo* and *in Vitro* Differences in DNA DSBs in Mouse Lymphocytes

There was no significant difference found in DNA DSBs between the *in vivo* and *in vitro* groups, regardless of whether 1 or 2 Gy of irradiation was applied (Table 1, *p* > 0.05). The results of *in vitro* irradiation may reflect the DNA damage induced after *in vivo* radiation exposure; therefore, the dose-response curves of *in vitro* irradiation may reflect DNA damage after *in vivo* radiation exposure.

After mouse lymphocytes were exposed to radiation *in vivo* and *in vitro* and the lymphocytic DNA DSBs were determined by the comet assay. As shown in Table 1, no significant difference was found between the two groups. Therefore, we were able to describe the dose-response curve after *in vitro* radiation, which reflected the DNA damage after *in vivo* radiation exposure.

### 2.3. DNA DSBs in Human Lymphocytes Induced by Radiation

**Figure 2:**
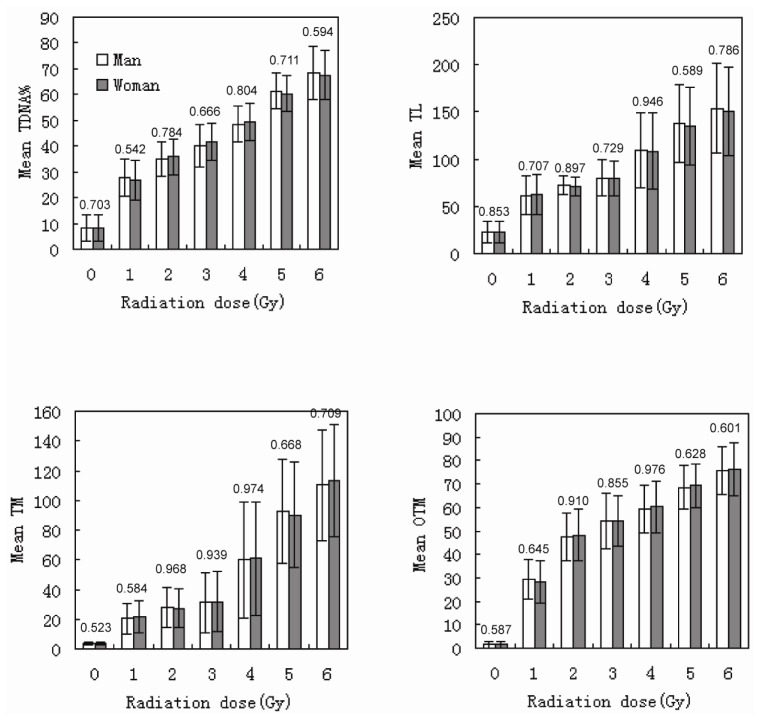
Variations in tail DNA (TDNA)%, tail length (TL), tail moment (TM), and olive tail moment (OTM) between male and female donors (the numbers above the column diagrams are *p* values between the male and female groups; error bars: ±1.00 SD).

**Figure 3:**
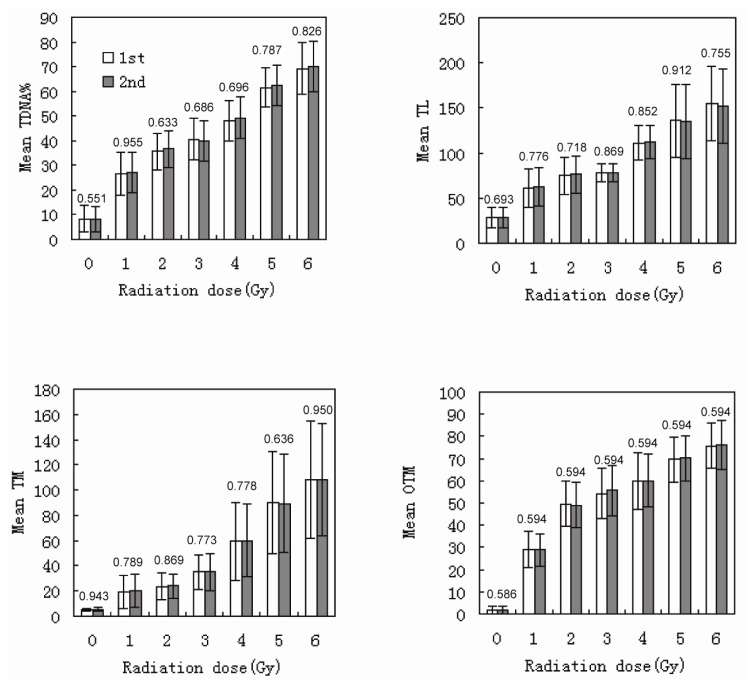
Variations in TDNA%, TL, TM, and OTM between the first and second experiments (the numbers above the column diagrams are *p* values between the first and second experiments; error bars: ±1.00 SD).

The DNA DSBs in human lymphocytes were induced by irradiation with ^137^Cs γ-rays at a dose of 0–5 Gy. The DNA fragments migrated to the positive pole during electrophoresis, and a “comet” was produced. Two donors were selected for this study, and the variation between the two donors was analyzed (Figure 2). Additionally, all of the experiments were repeated once, and the variation between the two experiments was analyzed. The results are shown in Figure 3. Because there were no significant differences between the results for female and male donors, we combined their data for the statistical analysis. The results of olive tail moment (OTM) are shown in Table 2.

The traditional cytogenetic methods for radiation biodosimetry include chromosomal aberration, micronuclei, premature chromosome condensation (PCC), and translocation analysis by fluorescent *in situ* hybridization (FISH) [4]. All of these methods have been used in analyses of radiation accidents. The chromosome aberration assay is considered the “gold standard” for radiation biodosimetry, but it is too time-consuming to facilitate a rapid-response analysis because lymphocyte culture requires approximately 50 h. Thus, rapid and sensitive methods are needed to monitor the DNA damage induced by ionizing radiation.

### 2.4. Dose- and Time-Response Relationship of DNA DSBs

**Figure 4:**
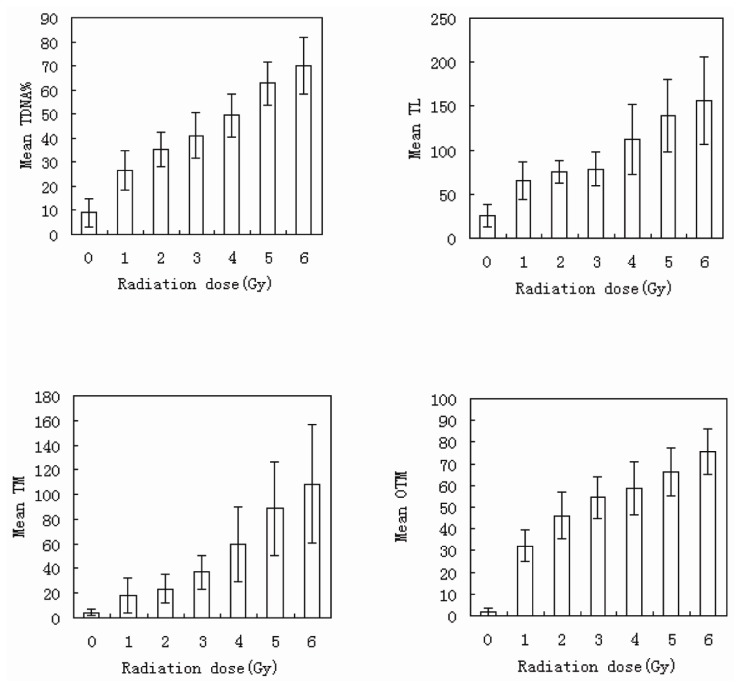
The column diagrams and error bars for TDNA%, TL, TM, and OTM detected immediately after irradiation by the comet assay (error bars: ±1.00 SD).

**Figure 5:**
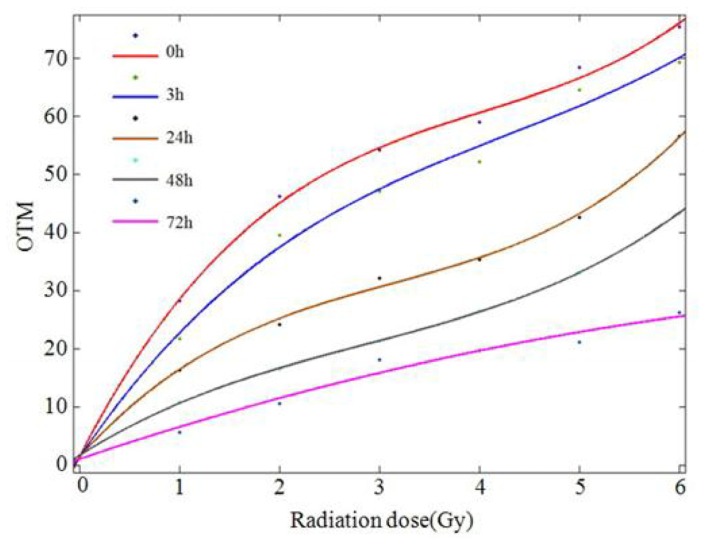
Dose-response curves of OTM obtained from the comet assay within 72 h after irradiation. [Dose-effect equations for different time points are shown below: “*y*” is the observed value of OTM; “*x*” is the radiation dose (Gy)]. Fit 1: *y*_0h_ = 0.5789*x*^3^ − 6.968*x*^2^ + 33.37*x* + 1.615 (*R*^2^ = 0.9979, *p* = 0.000); Fit 2: *y*_3h_ = 0.3397*x*^3^ − 4.364*x*^2^ + 25.4*x* + 1.43 (*R*^2^ = 0.9939, *p* = 0.000); Fit 3: *y*_24h_ = 0.4908*x*^3^ − 4.614*x*^2^ + 19.16*x* + 1.489 (*R*^2^ = 0.998, *p* = 0.000); Fit 4: *y*_48h_ = 0.2647*x*^3^ − 2.257*x*^2^ + 10.95*x* + 1.76 (*R*^2^ = 0.9999, *p* = 0.000); Fit 5: *y*_72h_ = 0.002222*x*^3^ − 0.304*x*^2^ + 5.818*x* + 1.156 (*R*^2^ = 0.9777, *p* = 0.000).

**Figure 6:**
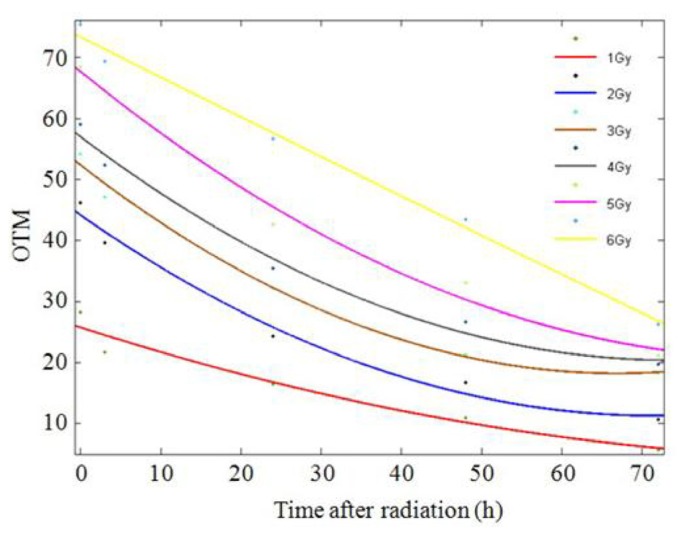
Time-response curves of OTM obtained from the comet assay within 72 h after irradiation. [Time-effect equations for different radiation doses are shown below: “*y*” is the observed value of OTM; “*x*” is the time after irradiation (h)]. Fit 1: *y*_1Gy_ = 0.002119*x*^2^ − 0.4259*x* + 25.73 (*R*^2^ = 0.9537, *p* = 0.000); Fit 2: *y*_2Gy_ = 0.006418*x*^2^ − 0.918*x* + 44.1 (*R*^2^ = 0.9847, *p* = 0.000); Fit 3: *y*_3Gy_ = 0.007615*x*^2^ − 1.019*x* + 52.29 (*R*^2^ = 0.9914, *p* = 0.000); Fit 4: *y*_4Gy_ = 0.006759*x*^2^ − 0.9946*x* + 56.95 (*R*^2^ = 0.9879, *p* = 0.000); Fit 5: *y*_5Gy_ = 0.006077*x*^2^ − 1.069*x* + 67.62 (*R*^2^ = 0.9896, *p* = 0.000); Fit 6: *y*_6Gy_ = 0.0001785*x*^2^ − 0.6587*x* + 73.31 (*R*^2^ = 0.9928, *p* = 0.000).

**Figure 7:**
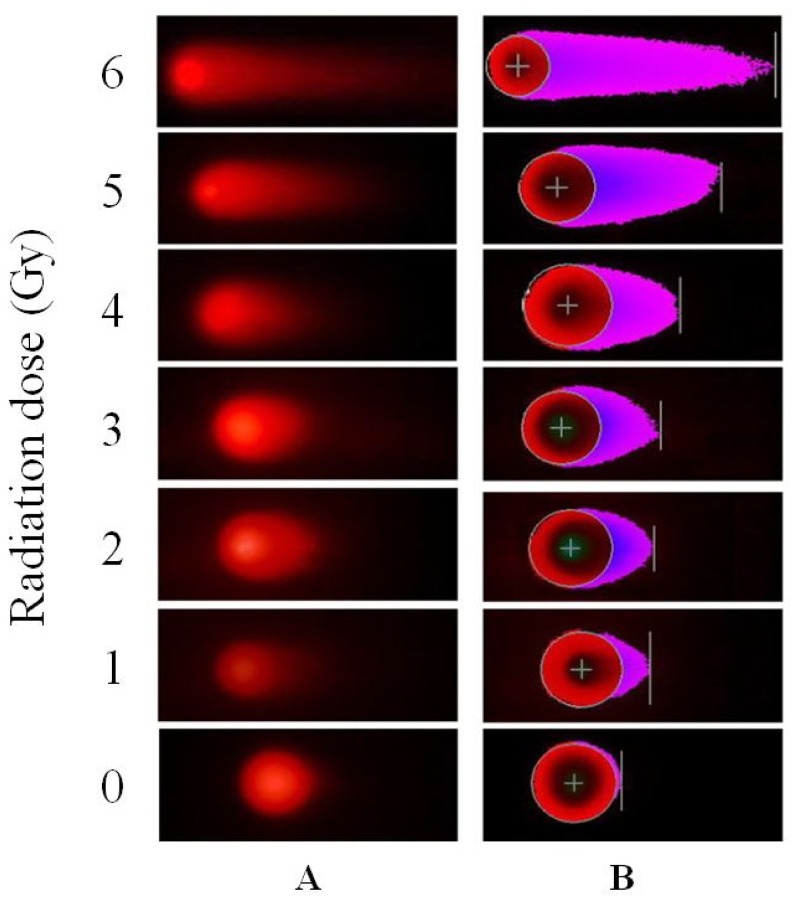
Comet images of lymphocytes after irradiation by γ-rays. (**A**): Original comet images; and (**B**): Comet images analyzed by CASP (CASP, Wroclaw, Poland).

**Figure 8:**
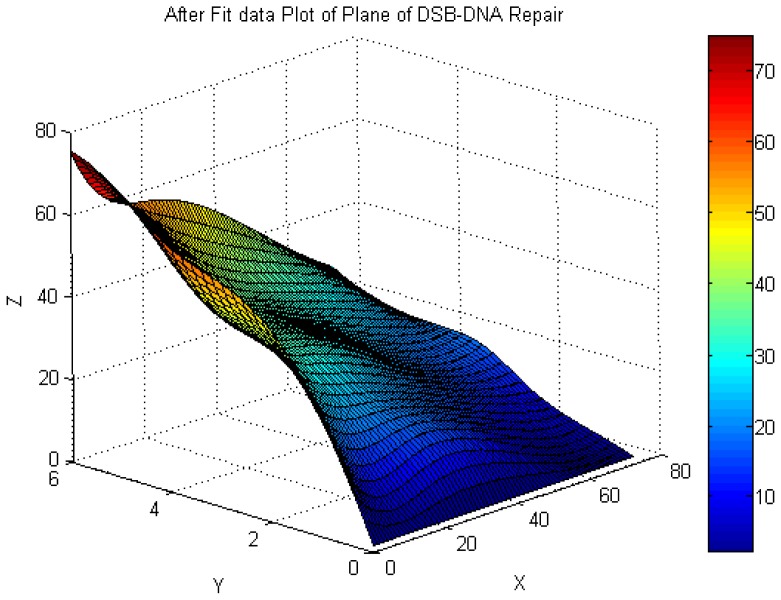
The three-dimensional plane model obtained by combining the dose- and time-response relationship of OTM derived from the comet assay within 72 h after irradiation [*Y* axis: radiation dose (Gy); *X* axis: time after irradiation (h); *Z* axis: value of OTM].

No significant difference was found between the male and female donors, and the same results were found between the two experiments; therefore, we pooled all of the data into one database to describe the dose-response relationship. The values of tail DNA (TDNA)%, tail length (TL), tail moment (TM), and OTM increased with increasing radiation dose, demonstrating a clear dose-response relationship. A comparison of the means of two optional groups revealed that the *p* values were all less than 0.01. The column diagrams and error bars for TDNA%, TL, TM, and OTM are shown in Figure 4. Curve-fitting was performed for OTM. The dose- and time-response curves are shown in Figures 5 and 6. Comet images of lymphocytes after irradiation are shown in Figure 7. The three-dimensional plane model was obtained when combining the dose- and time-response relationship of OTM obtained from the comet assay within 72 h after irradiation (Figure 8).

Linear and linear-quadratic relationships are generally used for biodosimetry. It is easy to calculate and estimate the radiation dose using linear and quadratic equations, but it is more difficult to calculate the radiation dose using polynomial functions. However, polynomial functions can be used as automated biodosimetry tools because they can be calculated easily using software [19,20]. In the present study, the polynomial function relationship was selected because the *R_2_* values (which represent goodness of fit) of the equations were better than those of linear and quadratic curves.

Comet assay research has shown clear dose-response relationships after irradiation in Chinese hamster ovary (CHO) cells [21], tumor cells [22,23], germ cells [7], and human lymphocytes [24]. Singh [25] detected human lymphocyte DNA DSBs and DNA single-strand breaks (SSBs) using the comet assay after irradiation with X-rays at 0.031–0.25 Gy (DNA SSBs) and 0.125–1 Gy (DNA DSBs). The results showed that the length of DNA migration and the radiation dose fit a linear relationship (*p* < 0.05 for both, *R*^2^ = 0.77 and *R*^2^ = 0.51 for DSBs and SSBs, respectively). These findings indicated a clear dose-response relationship over a small dose range (less than 1 Gy).

However, there are disadvantages of the comet assay in radiation biodosimetry. For example, the repair of DNA DSBs may influence the dose assessment results derived from the comet assay because in real-life situations, victims of radiation exposure are likely to be tested at various times after the exposure has occurred. Therefore, DNA repair kinetics after irradiation should always be assessed by the comet assay [26].

DNA DSBs can also be identified and quantified *in situ* by detecting the γ-H2AX foci formed at DNA break sites. Counting γ-H2AX foci is a sensitive method for determining irradiation-induced DSBs [27]. Because the presence of γ-H2AX is dependent on the DNA repair process, these foci can also be observed within several days of radiation exposure. Strong γ-H2AX induction was observed in blood lymphocytes following mini-pig total body irradiation in a recent study. Significant responses were detected 3 days after exposure to 1.8 Gy and 1 week after exposures to 3.8 and 5 Gy, with residual γ-H2AX foci proportional to the initial radiation doses [28].

With the use of traditional cytogenetic methods (e.g., chromosome aberration or micronucleus assays), some chromosome aberrations (dicentric, ring, and acentric chromosomes) or micronuclei may remain in lymphocytes 72 h or longer after irradiation. In our experiments, radiation may have induced altered chromatin metabolism leading to a significant number of temporary DSBs, that are not labeled with γ-H2AX, yet detected as DSBs by the neutral comet assay. Additionally, apoptotic cells were not included in the comet cell scoring in the present study.

In this study, we assessed the dose- and time-response relationship of DNA DSBs using the comet assay within 72 h after 1–6 Gy irradiation. To assess the potential use of the comet assay in radiation biodosimetry, the DNA repair kinetics should be considered in the dose-response curve. Therefore, we combined the dose-effect curve and the DNA repair kinetics into one model to design the three-dimensional plane model using the 3D difference calculation of the least squares method. This model indicated that we successfully found a suitable mathematical model for *in vitro* DNA DSB repair of human lymphocytes. Analysis software can be designed using this 3D plane model and used to estimate the radiation dose within 72 h in the dose range of 1–6 Gy. Different parameters may present different dose-response relationships at different doses or dose rate ranges. We focused on the dose-response relationship of DNA DSBs in the high dose range (>1 Gy). We will evaluate dose-response relationships by the comet assay following low-dose radiation exposures (<1 Gy) in future studies.

## 3. Experimental Section

### 3.1. Mice, Reagents, and Equipment

Eighteen male Chinese Kunming mice were purchased from the animal center of the Chinese Academy of Medical Science (age: 6–7 weeks; weight: 20 ± 2 g). The mice were randomly divided into the following three groups: the control, 1 Gy, and 2 Gy groups.

Normal-melting-point agarose was obtained from Biowest Co. (Kansas, MO, USA), and low-melting-point agarose was obtained from Promega Co. (Madison, WI, USA). Tris–HCl, dimethyl sulfoxide (DMSO), and Triton X-100 were obtained from Sigma (St. Louis, MO, USA). Lymphocyte separation medium (Lymphoprep) was obtained from Axis-Shield (Axis-Shield PoC, Oslo, Norway). The horizontal-strip electrophoresis apparatus was obtained from Bio-Rad (Bio Rad Laboratories Inc, Hercules, CA, USA). The ^137^Cs radiation source was obtained from Atomic Energy Co. (Atomic Energy of Canadian Inc., Mississauga, ON, Canada). A Nikon 90i fluorescence microscope was obtained from Nikon Co. (Tokyo, Japan). The comet slides were obtained from Biocomet Co. (Biocomet, Nanning, Guangxi, China). The digital imaging system was purchased from Union Science Inc. (Cherry Hill, NJ, USA). The Sanyo MCO-20AIC CO_2_ incubator was purchased from Sanyo (Sakata, Japan).

### 3.2. Collection of Blood Samples and Selection of the Radiation Exposure Groups

A volume of 0.2 mL of peripheral blood was collected from each mouse via the orbital sinus using a glass capillary, and all groups were anticoagulated with heparin. Then, the blood (*in vitro*) and corresponding mouse (*in vivo*) were exposed at the same time to ^137^Cs at 1 or 2 Gy. Peripheral blood was collected from each mouse for the second time after irradiation (*in vivo*).

One healthy adult man and one healthy adult woman were selected for this study. Neither adult had any history of smoking, excessive alcohol consumption, or exposure to radiation or any other industrial poison. Peripheral blood was obtained from each donor in a 5 mL-tube containing heparin sodium. The blood was then divided into eight parts for irradiation at various doses. The radiation doses were 0 (control group), 1, 2, 3, 4, 5, and 6 Gy. The absorbed dose rate was 0.8451 Gy/min. The comet assay was performed immediately after each exposure. To assess the time-response relationship of DNA repair, 7 mL of peripheral blood was required; then, each blood sample was divided equally into 7 parts for irradiation at various doses (0, 1, 2, 3, 4, 5, and 6 Gy). The irradiated samples were then divided equally into four parts for the detection of DNA DSBs at different time points (3, 24, 48, and 72 h after irradiation) to assess the DNA repair kinetics. The blood samples were incubated in RPMI-1640 medium containing 20% bovine calf serum at 37 °C in a 5% CO_2_ incubator for 3, 24, 48, and 72 h for further comet assay. Prior to the study, written informed consent was obtained from all of the subjects. Because 40 min were required to prepare the lymphocytes in agarose for lysis from whole blood after irradiation, resulting in some repair, this time point could not be considered *t* = 0 h. The *t* = 0 h group consisted of lymphocytes isolated from whole blood and embedded in low-melting-point agarose on the comet slides before irradiation. The comet slides were immersed in cold fresh lysis solution immediately after irradiation. Using this procedure, we aimed to detect the DNA DSBs that were reflective of the initial damage after exposure. The Institutional Animal Care and Use Committee of Peking Union Medical College (PUMC) approved all of the experimental procedures used in this study.

### 3.3. Lymphocyte Isolation and Comet Assay

A volume of 0.15 mL of whole blood was carefully layered onto the lymphocyte separation medium in a centrifugation tube at a ratio of 1:2. A gray layer of lymphocytes appeared between the blood plasma and the lymphoprep after centrifugation for 2 min at 3500 rpm. The lymphocytes were then carefully transferred to a 1.5-mL Eppendorf tube containing 1.2 mL of phosphate-buffered saline (PBS, 0.1 M) and were centrifuged for 5 min at 2000 rpm. The lymphocytes were washed twice with PBS. The cells were suspended in PBS, and the density was adjusted to 5–6 × 10^4^/mL. The cell viability, as assessed by the Trypan blue test, was 98%, and the lymphocyte suspension was then stored in a refrigerator at 4 °C.

The comet assay was performed under neutral conditions, as described by Banath *et al.* (1998) [29], but with a slight modification; specifically, special comet slides were used rather than general slides. There are gaps in comet slides to contain the agarose. Furthermore, much less agarose was used in the procedure than originally described by Banath, yielding much thinner gel agarose to enable clearer viewing under the fluorescence microscope. First, the comet slides was coated with 100 μL of normal-melting-point agarose (0.075%); then, once the first agarose layer was coagulated, a mixture of 75 μL of low-melting-point agarose (0.075%) and 25 μL of lymphocyte suspension was applied as the second layer. The comet slides were immersed in cold fresh lysis solution (2.5 M NaCl, 1% *N*-sodium lauryl sarcosinate, 30 mM Na_2_EDTA, 10 mM Tris, 1% Triton X-100, 10% DMSO) for 1.5 h at 4 °C. After lysis, they were placed in buffer for 20 min in a horizontal electrophoresis tank pre-filled with cold fresh Tris-borate-EDTA buffer (TBE) to loosen the tight double-helical structure of DNA for electrophoresis. Electrophoresis was then performed at 200 mA for 20 min in TBE buffer at room temperature. The slides were then rinsed twice with distilled water and were stained with ethidium bromide (2 μg/mL). All of the above procedures were performed in the dark to avoid supernumerary DNA damage. The comets were viewed using a Nikon 90i fluorescence microscope, and images of 100 comets were collected for each group using a digital imaging system. Cells that overlapped were not counted. All of the comet images were analyzed using CASP software (CASP, Wroclaw, Poland) [30], and the percentage of DNA in the comet tail (TDNA%), TL, TM, and the OTM were recorded to characterize the lymphocytic DNA damage. All of the experiments were repeated once, and the variation between experiments was analyzed.

### 3.4. Statistical Analysis

A paired-samples *t* test was used to compare the differences in DNA DSBs between the *in vivo* and *in vitro* groups. Statistical analyses were performed using one-way analysis of variance (ANOVA), and significance was assumed if *p* < 0.05. Matlab software, version 7.8 (MathWorks, Natick, MA, USA), was used to fit the dose- and time-response (repair) curves, and the two curves were then combined to design the three-dimensional plane model using the 3D difference calculation performed using the least squares method.

## 4. Conclusions

We created a model for the repair of DNA damage within 72 h after 1–6 Gy irradiation. Curves of DNA DSBs and DNA repair after different doses of radiation showed a linear relationship; therefore, the 3D plane model may be useful for biodosimetry analysis of radiation-induced DNA damage. Further studies should be performed to increase the practical value of the model in the estimation of biological radiation doses.

## Figures and Tables

**Table 1:** Comparison between the *in vivo* and *in vitro* DNA double-strand breaks (DSBs) in mouse lymphocytes (*χ̄* ± SD).

Group	*N*	Control			1 Gy			2 Gy		
		
		*in vivo*	*in vitro*	*p*	*in vivo*	*in vitro*	*p*	*in vivo*	*in vitro*	*p*
Tail DNA (TDNA) %	6	5.8 ± 1.8	61 ± 2.1	0.27	31 ± 11.9	33 ± 10.7	0.16	39 ± 6.6	41 ± 7.8	0.40
Tail length (TL) (pix)	6	18.1 ± 5.2	19.9 ± 6.3	0.43	68.1 ± 27.1	70.1 ± 29.6	0.36	77.2 ± 15.2	79.4 ± 13.7	0.71
Tail moment (TM)	6	2.1 ± 0.6	2.6 ± 0.8	0.29	24.1 ± 9.2	25.1 ± 10.8	0.23	31.6 ± 13.2	34.7 ± 10.9	0.10
Olive tail moment (OTM)	6	1.9 ± 0.6	1.7 ± 0.8	0.18	13.6 ± 7.9	14.4 ± 6.6	0.11	18.7 ± 6.46	19.2 ± 7.1	0.13

Note: pix: pixel, unit of TL.

**Table 2:** DNA DSBs olive tail moment (OTM) in lymphocytes after irradiation with various doses within 72 h (*χ̄* ± SD).

Group	control	1 Gy	2 Gy	3 Gy	4 Gy	5 Gy	6 Gy
0 h	1.6 ± 1.6	28.3 ± 8.2	46.2 ± 10.1	54.2 ± 11.7	59.0 ± 10.2	68.5 ± 9.8	75.4 ± 10.1
3 h	1.6 ± 1.7	21.7 ± 6.3	39.6 ± 13.6	47.2 ± 8.7	52.3 ± 7.2	64.6 ± 8.7	69.3 ± 8.3
24 h	1.6 ± 0.7	16.5 ± 8.0	24.3 ± 10.1	32.2 ± 13.9	35.4 ± 8.0	42.7 ± 10.5	56.7 ± 20.7
48 h	1.7 ± 1.5	10.9 ± 6.2	16.6 ± 11.7	21.3 ± 7.6	26.6 ± 9.4	33.1 ± 7.5	43.4 ± 14.3
72 h	1.7 ± 1.9	5.7 ± 5.2	10.6 ± 9.7	18.2 ± 13.2	19.7 ± 9.3	21.2 ± 15.1	26.3 ± 18.0
